# Di-μ-iodido-bis­[(diethyl ether-κ*O*)(η^5^-1,3-di-*tert*-butyl­cyclo­penta­dien­yl)ytterbium(II)]

**DOI:** 10.1107/S1600536807066871

**Published:** 2007-12-21

**Authors:** Madeleine Schultz

**Affiliations:** a School of Physical and Chemical Sciences, Queensland University of Technology, Brisbane, Queensland 4001, Australia

## Abstract

The half-sandwich title compound, [Yb_2_(C_13_H_21_)_2_I_2_(C_4_H_10_O)_2_], crystallizes as a centrosymmetric dimer. The Yb atom is coordinated in a three-legged piano-stool geometry by a cyclo­penta­dienyl ring, two I anions and the O atom of a diethyl ether mol­ecule. The central Yb_2_I_2_ core is an approximate square.

## Related literature

For related structures, see: Constantine *et al.* (1996 ▶); Trifonov *et al.* (2003 ▶). For related chemistry, see: Schultz *et al.* (2000 ▶); Schumann *et al.* (1993 ▶).
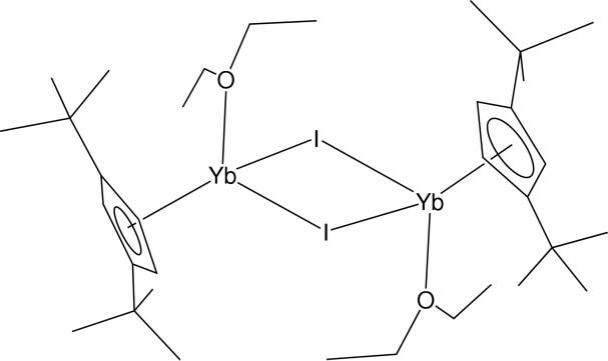


## Experimental

### 

#### Crystal data

[Yb_2_(C_13_H_21_)_2_I_2_(C_4_H_10_O)_2_]
*M* *_r_* = 1102.72
Monoclinic, 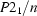

*a* = 13.7190 (3) Å
*b* = 11.1690 (1) Å
*c* = 14.4440 (3) Å
β = 112.800 (1)°
*V* = 2040.28 (6) Å^3^
*Z* = 2
Mo *K*α radiation
μ = 6.10 mm^−1^
*T* = 160 K
0.40 × 0.30 × 0.15 mm

#### Data collection

Bruker SMART 1K CCD diffractometer
Absorption correction: multi-scan (*XPREP*; Sheldrick, 1995 ▶) *T*_min_ = 0.192, *T*_max_ = 0.401
8321 measured reflections
2928 independent reflections
2679 reflections with *I* > 2σ(*I*)
*R*_int_ = 0.033
θ_max_ = 23.3°

#### Refinement

*R*[*F* ^2^ > 2σ(*F* ^2^)] = 0.026
*wR*(*F* ^2^) = 0.059
*S* = 1.09
2928 reflections
181 parameters
H-atom parameters constrained
Δρ_max_ = 0.92 e Å^−3^
Δρ_min_ = −1.11 e Å^−3^

### 

Data collection: *SMART* (Siemens, 1995 ▶); cell refinement: *SAINT* (Siemens, 1995 ▶); data reduction: *SAINT*; program(s) used to solve structure: *SIR92* (Altomare *et al.*, 1994 ▶); program(s) used to refine structure: *SHELXL97* (Sheldrick, 1997 ▶); molecular graphics: *XP* in *SHELXTL* (Sheldrick, 1998 ▶); software used to prepare material for publication: *SHELXL97*.

## Supplementary Material

Crystal structure: contains datablocks I, global. DOI: 10.1107/S1600536807066871/tk2232sup1.cif

Structure factors: contains datablocks I. DOI: 10.1107/S1600536807066871/tk2232Isup2.hkl

Additional supplementary materials:  crystallographic information; 3D view; checkCIF report

## Figures and Tables

**Table:** Cp is the calculated centroid of atoms C1–C5.

Yb1—Cp	2.37
Yb1—O1	2.387 (5)
Yb1—I1	3.0848 (4)
Yb1—I1^i^	3.0961 (5)

**Table:** 

I1—Yb1—Cp	127
O1—Yb1—Cp	124
I1—Yb1—Cp^i^	115
O1—Yb1—I1	96.34 (11)
O1—Yb1—I1^i^	97.98 (13)
I1—Yb1—I1^i^	88.78 (1)
Yb1—I1—Yb1^i^	91.22 (1)

Symmetry code: (i) 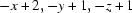.

**Fig. 1.**
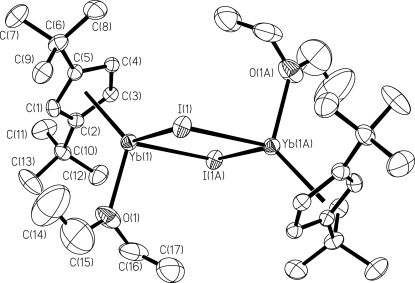
A view of the dimer (I) showing the atom labelling scheme. Hydrogen atoms have been omitted for clarity and displacement ellipsoids are drawn at the 50% probability level. Symmetry operation i: -x + 2, -y + 1, -z + 1.

## supplementary crystallographic information

### Comment

The title compound (I) was formed in an attempt to prepare the metallocene of the substituted cyclopentadienyl ligand by stirring one equivalent of the magnesocene (η^5^-1,3-(Me_3_C)_2_C_5_H_3_)_2_Mg with YbI_2_ in diethyl ether. The reaction can also be performed using half an equivalent of magnesocene. The inital yellow-green slurry forms a deep green solution after stirring for two hours at room temperature. The green color has previously been associated with formation of an ytterbocene. However, upon filtration a thermochroic solution results that is bright-green below -30°C but becomes orange-brown upon warming to room temperature. The bright-orange crystals that form at low temperature do not redissolve in diethyl ether and are insoluble in hydrocarbon solvents. Use of THF as solvent for the reaction leads to transfer of two cyclopentadienide rings to the metal and formation of the known THF adduct [1,3-(Me_3_C)_2_C_5_H_3_]_2_Yb(THF) (Schumann *et al.*, 1993).

The structure of (I) is centrosymmetric about a square Yb_2_I_2_ core, Fig. 1 & Table 1. There is gross thermal motion in one ethyl group of the diethyl ether ligand, affecting the thermal parameters of C(14) in particular, but this does not adversely impact the quality of the core of the structure. The distances and angles fall within normal ranges for Yb(II) in 6-coordination. (Schultz *et al.*, 2000)

The structure is similar to those of the published dimers {(Me_5_C_5_)Yb(THF)_2_-µ-I}_2_, {(Me_5_C_5_)Yb(dme)-µ-I}_2_, (Constantine *et al.*, 1996) and {[Me_4_[SiMe_2_NH(CMe_3_)]C_5_]Yb(THF)_2_-µ-I}_2_ (Trifonov *et al.*, 2003), in which the Yb(II) is 7-coordinate.

### Experimental

The magnesocene [1,3-(Me_3_C)_2_C_5_H_3_]_2_Mg (0.5 g, 1.3 mmol) was weighed into a round-bottomed flask equipped with a magnetic stirrer bar. YbI_2_ (0.56 g, 1.3 mmol) was added under a flow of N_2_. Et_2_O was added and the slurry was stirred at room temperature. After 1 h, the solution was green in color. After 3 h, the solution was filtered off the grey solid which appeared to contain unreacted magnesocene by ^1^H NMR spectroscopy. The volume of the green solution was reduced under reduced pressure; the solid that was deposited on the sides of the flask during this procedure was orange. Cooling to -40°C resulted in the formation of clear orange crystals which were insoluble in OEt_2_, hot toluene or C_6_D_6_. The crystals turn brown at 130°C, and black at 230°C, but do not melt to 330°C. Analysis. Found: C 38.2, H 5.96%. C_17_H_31_IOYb requires C 37.0, H 5.67%. The insolubility of the dimer prevented recrystallization or the obtention of an NMR spectrum. Sublimation of the dimer did not lead to the formation of the ytterbocene. The ether adduct of magnesium iodide can be obtained as a first crop of colorless crystals from the mother liquor before crystallization of the orange product, and unreacted [1,3-(Me_3_C)_2_C_5_H_3_]_2_Mg can be crystallized from the mother liquor after all of the product has crystallized from the solution.

### Refinement

All H atoms were positioned geometrically and allowed to ride on their parent atoms with C—H distances in the range 0.93–0.97 Å, and with *U*_iso_(H) = 1.5 *U*_eq_(C) for methyl-H atoms and *U*_iso_(H) = 1.2 *U*_eq_(C) for other atoms. The maximum and minimum residual electron density peaks of 0.92 and -1.11 e Å^-3^ were located 0.94 and 0.94 Å, respectively from the H15A and Yb atoms.

### Crystal data

**Table:**

[Yb_2_(C_13_H_21_)_2_I_2_(C_4_H_10_O)_2_]	*F*_000_ = 1056
*M**_r_* = 1102.72	*D*_x_ = 1.795 Mg m^−^^3^
Monoclinic, *P*2_1_/*n*	Melting point: 330 K
Hall symbol: -P 2yn	Mo *K*α radiation λ = 0.71073 Å
*a* = 13.7190 (3) Å	Cell parameters from 6017 reflections
*b* = 11.1690 (1) Å	θ = 2.0–23.3º
*c* = 14.4440 (3) Å	µ = 6.10 mm^−^^1^
β = 112.800 (1)º	*T* = 160 K
*V* = 2040.28 (6) Å^3^	Plate-like, orange
*Z* = 2	0.40 × 0.30 × 0.15 mm

### Data collection

**Table:**

Bruker SMART 1K CCD diffractometer	2928 independent reflections
Radiation source: fine-focus sealed tube	2679 reflections with *I* > 2σ(*I*)
Monochromator: graphite	*R*_int_ = 0.033
*T* = 161(2) K	θ_max_ = 23.3º
ω scans	θ_min_ = 1.7º
Absorption correction: multi-scan(XPREP; Sheldrick, 1995)	*h* = −15→15
*T*_min_ = 0.192, *T*_max_ = 0.401	*k* = −12→7
8321 measured reflections	*l* = −15→16

### Refinement

**Table:**

Refinement on *F*^2^	Secondary atom site location: difference Fourier map
Least-squares matrix: full	Hydrogen site location: inferred from neighbouring sites
*R*[*F*^2^ > 2σ(*F*^2^)] = 0.026	H-atom parameters constrained
*wR*(*F*^2^) = 0.059	*w* = 1/[σ^2^(*F*_o_^2^) + (0.0144*P*)^2^ + 9.3362*P*] where *P* = (*F*_o_^2^ + 2*F*_c_^2^)/3
*S* = 1.09	(Δ/σ)_max_ = 0.001
2928 reflections	Δρ_max_ = 0.92 e Å^−^^3^
181 parameters	Δρ_min_ = −1.11 e Å^−^^3^
Primary atom site location: structure-invariant direct methods	Extinction correction: none

### Special details

**Table:**

Geometry. All e.s.d.'s (except the e.s.d. in the dihedral angle between two l.s. planes) are estimated using the full covariance matrix. The cell e.s.d.'s are taken into account individually in the estimation of e.s.d.'s in distances, angles and torsion angles; correlations between e.s.d.'s in cell parameters are only used when they are defined by crystal symmetry. An approximate (isotropic) treatment of cell e.s.d.'s is used for estimating e.s.d.'s involving l.s. planes.
Refinement. Refinement of *F*^2^ against ALL reflections. The weighted *R*-factor *wR* and goodness of fit *S* are based on *F*^2^, conventional *R*-factors *R* are based on *F*, with *F* set to zero for negative *F*^2^. The threshold expression of *F*^2^ > σ(*F*^2^) is used only for calculating *R*-factors(gt) *etc*. and is not relevant to the choice of reflections for refinement. *R*-factors based on *F*^2^ are statistically about twice as large as those based on *F*, and *R*- factors based on ALL data will be even larger.

### Fractional atomic coordinates and isotropic or equivalent isotropic displacement parameters (Å^2^)

**Table:**

	*x*	*y*	*z*	*U*_iso_*/*U*_eq_	
Yb1	0.848794 (19)	0.48479 (2)	0.519907 (18)	0.02631 (9)	
I1	0.92348 (3)	0.43514 (4)	0.34774 (3)	0.03074 (12)	
O1	0.7760 (4)	0.6781 (4)	0.4630 (3)	0.0524 (13)	
C1	0.6879 (4)	0.3797 (5)	0.5476 (4)	0.0257 (13)	
H1	0.6204	0.4075	0.5090	0.031*	
C2	0.7543 (4)	0.4259 (5)	0.6424 (4)	0.0262 (13)	
C3	0.8486 (4)	0.3596 (5)	0.6738 (4)	0.0231 (12)	
H3	0.9067	0.3707	0.7336	0.028*	
C4	0.8410 (4)	0.2731 (5)	0.5998 (4)	0.0238 (12)	
H4	0.8931	0.2183	0.6027	0.029*	
C5	0.7402 (4)	0.2844 (5)	0.5203 (4)	0.0240 (12)	
C6	0.6922 (5)	0.2019 (5)	0.4301 (4)	0.0310 (14)	
C7	0.6089 (5)	0.1221 (6)	0.4471 (5)	0.0444 (17)	
H7A	0.5548	0.1715	0.4538	0.067*	
H7B	0.6421	0.0758	0.5072	0.067*	
H7C	0.5779	0.0692	0.3909	0.067*	
C8	0.7764 (5)	0.1225 (6)	0.4175 (5)	0.0416 (16)	
H8A	0.7437	0.0681	0.3627	0.062*	
H8B	0.8119	0.0780	0.4781	0.062*	
H8C	0.8267	0.1714	0.4037	0.062*	
C9	0.6382 (5)	0.2726 (6)	0.3338 (4)	0.0351 (15)	
H9A	0.6895	0.3210	0.3211	0.053*	
H9B	0.5848	0.3232	0.3406	0.053*	
H9C	0.6061	0.2183	0.2788	0.053*	
C10	0.7263 (5)	0.5216 (5)	0.7035 (4)	0.0314 (14)	
C11	0.6980 (7)	0.4584 (7)	0.7850 (6)	0.058 (2)	
H11A	0.6863	0.5174	0.8280	0.088*	
H11B	0.7551	0.4069	0.8242	0.088*	
H11C	0.6350	0.4117	0.7536	0.088*	
C12	0.8200 (5)	0.6051 (6)	0.7574 (5)	0.0460 (17)	
H12A	0.8400	0.6453	0.7087	0.069*	
H12B	0.8787	0.5589	0.8015	0.069*	
H12C	0.8001	0.6631	0.7958	0.069*	
C13	0.6325 (6)	0.5959 (8)	0.6373 (6)	0.063 (2)	
H13A	0.6493	0.6345	0.5860	0.095*	
H13B	0.6166	0.6553	0.6774	0.095*	
H13C	0.5722	0.5447	0.6067	0.095*	
C14	0.6291 (11)	0.6154 (14)	0.3336 (11)	0.168 (8)	
H14A	0.5707	0.6418	0.2749	0.251*	
H14B	0.6030	0.5790	0.3797	0.251*	
H14C	0.6701	0.5580	0.3145	0.251*	
C15	0.6885 (9)	0.7070 (13)	0.3767 (9)	0.117 (4)	
H15A	0.6459	0.7653	0.3939	0.140*	
H15B	0.7129	0.7443	0.3289	0.140*	
C16	0.8367 (7)	0.7862 (7)	0.5083 (6)	0.061 (2)	
H16A	0.8818	0.7693	0.5776	0.073*	
H16B	0.7880	0.8491	0.5083	0.073*	
C17	0.9034 (7)	0.8298 (8)	0.4551 (7)	0.075 (3)	
H17A	0.9409	0.9003	0.4879	0.112*	
H17B	0.8592	0.8485	0.3868	0.112*	
H17C	0.9530	0.7687	0.4562	0.112*	

### Atomic displacement parameters (Å^2^)

**Table:**

	*U*^11^	*U*^22^	*U*^33^	*U*^12^	*U*^13^	*U*^23^
Yb1	0.02713 (15)	0.02691 (15)	0.02587 (15)	−0.00026 (11)	0.01133 (11)	0.00181 (11)
I1	0.0296 (2)	0.0388 (2)	0.0227 (2)	−0.00659 (17)	0.00885 (16)	−0.00354 (17)
O1	0.058 (3)	0.056 (3)	0.045 (3)	0.023 (3)	0.021 (2)	0.022 (3)
C1	0.019 (3)	0.033 (3)	0.021 (3)	0.002 (3)	0.004 (2)	0.002 (3)
C2	0.029 (3)	0.025 (3)	0.025 (3)	0.000 (3)	0.011 (3)	0.001 (3)
C3	0.025 (3)	0.025 (3)	0.017 (3)	−0.002 (2)	0.006 (2)	0.003 (2)
C4	0.023 (3)	0.024 (3)	0.024 (3)	0.000 (2)	0.009 (2)	0.002 (2)
C5	0.024 (3)	0.025 (3)	0.025 (3)	−0.002 (2)	0.011 (2)	−0.001 (2)
C6	0.035 (3)	0.028 (3)	0.025 (3)	−0.008 (3)	0.006 (3)	−0.005 (3)
C7	0.051 (4)	0.047 (4)	0.031 (3)	−0.021 (3)	0.010 (3)	−0.004 (3)
C8	0.047 (4)	0.034 (4)	0.039 (4)	0.000 (3)	0.012 (3)	−0.014 (3)
C9	0.036 (3)	0.042 (4)	0.024 (3)	−0.009 (3)	0.007 (3)	−0.005 (3)
C10	0.034 (3)	0.034 (3)	0.031 (3)	0.006 (3)	0.018 (3)	0.000 (3)
C11	0.090 (6)	0.046 (4)	0.067 (5)	−0.012 (4)	0.060 (5)	−0.010 (4)
C12	0.049 (4)	0.044 (4)	0.049 (4)	−0.013 (4)	0.023 (3)	−0.016 (3)
C13	0.057 (5)	0.071 (6)	0.056 (5)	0.035 (4)	0.015 (4)	−0.013 (4)
C14	0.122 (11)	0.171 (15)	0.141 (12)	−0.067 (11)	−0.024 (9)	0.081 (12)
C15	0.087 (8)	0.138 (12)	0.091 (8)	0.016 (8)	−0.004 (7)	0.041 (8)
C16	0.090 (6)	0.036 (4)	0.072 (5)	0.020 (4)	0.047 (5)	0.009 (4)
C17	0.087 (6)	0.065 (6)	0.096 (7)	0.017 (5)	0.060 (6)	0.014 (5)

### Geometric parameters (Å, °)

**Table:**

Yb1—Cp	2.37	C8—H8B	0.9600
Yb1—O1	2.387 (5)	C8—H8C	0.9600
Yb1—C3	2.627 (5)	C9—H9A	0.9600
Yb1—C2	2.650 (5)	C9—H9B	0.9600
Yb1—C4	2.651 (5)	C9—H9C	0.9600
Yb1—C1	2.663 (5)	C10—C13	1.519 (9)
Yb1—C5	2.690 (5)	C10—C12	1.533 (9)
Yb1—I1	3.0848 (4)	C10—C11	1.547 (9)
Yb1—I1^i^	3.0961 (5)	C11—H11A	0.9600
I1—Yb1^i^	3.0961 (5)	C11—H11B	0.9600
O1—C15	1.393 (10)	C11—H11C	0.9600
O1—C16	1.469 (9)	C12—H12A	0.9600
C1—C2	1.416 (8)	C12—H12B	0.9600
C1—C5	1.422 (8)	C12—H12C	0.9600
C1—H1	0.9300	C13—H13A	0.9600
C2—C3	1.405 (8)	C13—H13B	0.9600
C2—C10	1.526 (8)	C13—H13C	0.9600
C3—C4	1.413 (7)	C14—C15	1.306 (16)
C3—H3	0.9300	C14—H14A	0.9600
C4—C5	1.419 (7)	C14—H14B	0.9600
C4—H4	0.9300	C14—H14C	0.9600
C5—C6	1.522 (8)	C15—H15A	0.9700
C6—C9	1.519 (8)	C15—H15B	0.9700
C6—C8	1.521 (8)	C16—C17	1.486 (10)
C6—C7	1.543 (8)	C16—H16A	0.9700
C7—H7A	0.9600	C16—H16B	0.9700
C7—H7B	0.9600	C17—H17A	0.9600
C7—H7C	0.9600	C17—H17B	0.9600
C8—H8A	0.9600	C17—H17C	0.9600
			
I1—Yb1—Cp	127	C8—C6—C7	109.0 (5)
O1—Yb1—Cp	124	C5—C6—C7	108.3 (5)
I1—Yb1—Cp^i^	115	C6—C7—H7A	109.5
O1—Yb1—C3	129.45 (16)	C6—C7—H7B	109.5
O1—Yb1—C2	101.92 (16)	H7A—C7—H7B	109.5
C3—Yb1—C2	30.87 (17)	C6—C7—H7C	109.5
O1—Yb1—C4	150.96 (16)	H7A—C7—H7C	109.5
C3—Yb1—C4	31.06 (16)	H7B—C7—H7C	109.5
C2—Yb1—C4	51.34 (17)	C6—C8—H8A	109.5
O1—Yb1—C1	100.94 (17)	C6—C8—H8B	109.5
C3—Yb1—C1	50.62 (17)	H8A—C8—H8B	109.5
C2—Yb1—C1	30.92 (17)	C6—C8—H8C	109.5
C4—Yb1—C1	50.65 (17)	H8A—C8—H8C	109.5
O1—Yb1—C5	126.52 (17)	H8B—C8—H8C	109.5
C3—Yb1—C5	51.15 (17)	C6—C9—H9A	109.5
C2—Yb1—C5	51.49 (17)	C6—C9—H9B	109.5
C4—Yb1—C5	30.81 (16)	H9A—C9—H9B	109.5
C1—Yb1—C5	30.80 (16)	C6—C9—H9C	109.5
O1—Yb1—I1	96.34 (11)	H9A—C9—H9C	109.5
C3—Yb1—I1	134.10 (12)	H9B—C9—H9C	109.5
C2—Yb1—I1	153.51 (12)	C13—C10—C2	111.1 (5)
C4—Yb1—I1	105.83 (12)	C13—C10—C12	109.1 (6)
C1—Yb1—I1	126.53 (12)	C2—C10—C12	111.7 (5)
C5—Yb1—I1	102.10 (11)	C13—C10—C11	109.1 (6)
O1—Yb1—I1^i^	97.98 (13)	C2—C10—C11	108.3 (5)
C3—Yb1—I1^i^	87.89 (12)	C12—C10—C11	107.4 (5)
C2—Yb1—I1^i^	107.27 (12)	C10—C11—H11A	109.5
C4—Yb1—I1^i^	100.96 (11)	C10—C11—H11B	109.5
C1—Yb1—I1^i^	136.99 (11)	H11A—C11—H11B	109.5
C5—Yb1—I1^i^	131.77 (12)	C10—C11—H11C	109.5
I1—Yb1—I1^i^	88.782 (12)	H11A—C11—H11C	109.5
Yb1—I1—Yb1^i^	91.218 (11)	H11B—C11—H11C	109.5
C15—O1—C16	110.6 (8)	C10—C12—H12A	109.5
C15—O1—Yb1	128.2 (7)	C10—C12—H12B	109.5
C16—O1—Yb1	120.1 (4)	H12A—C12—H12B	109.5
C2—C1—C5	109.7 (5)	C10—C12—H12C	109.5
C2—C1—Yb1	74.0 (3)	H12A—C12—H12C	109.5
C5—C1—Yb1	75.6 (3)	H12B—C12—H12C	109.5
C2—C1—H1	125.2	C10—C13—H13A	109.5
C5—C1—H1	125.2	C10—C13—H13B	109.5
Yb1—C1—H1	117.0	H13A—C13—H13B	109.5
C3—C2—C1	106.6 (5)	C10—C13—H13C	109.5
C3—C2—C10	126.1 (5)	H13A—C13—H13C	109.5
C1—C2—C10	127.1 (5)	H13B—C13—H13C	109.5
C3—C2—Yb1	73.7 (3)	C15—C14—H14A	109.5
C1—C2—Yb1	75.1 (3)	C15—C14—H14B	109.5
C10—C2—Yb1	120.7 (4)	H14A—C14—H14B	109.5
C2—C3—C4	109.1 (5)	C15—C14—H14C	109.5
C2—C3—Yb1	75.5 (3)	H14A—C14—H14C	109.5
C4—C3—Yb1	75.4 (3)	H14B—C14—H14C	109.5
C2—C3—H3	125.4	C14—C15—O1	114.0 (11)
C4—C3—H3	125.4	C14—C15—H15A	108.7
Yb1—C3—H3	115.7	O1—C15—H15A	108.7
C3—C4—C5	108.3 (5)	C14—C15—H15B	108.7
C3—C4—Yb1	73.5 (3)	O1—C15—H15B	108.7
C5—C4—Yb1	76.1 (3)	H15A—C15—H15B	107.6
C3—C4—H4	125.8	O1—C16—C17	113.3 (6)
C5—C4—H4	125.8	O1—C16—H16A	108.9
Yb1—C4—H4	116.6	C17—C16—H16A	108.9
C4—C5—C1	106.3 (5)	O1—C16—H16B	108.9
C4—C5—C6	127.0 (5)	C17—C16—H16B	108.9
C1—C5—C6	126.4 (5)	H16A—C16—H16B	107.7
C4—C5—Yb1	73.1 (3)	C16—C17—H17A	109.5
C1—C5—Yb1	73.6 (3)	C16—C17—H17B	109.5
C6—C5—Yb1	123.9 (3)	H17A—C17—H17B	109.5
C9—C6—C8	108.5 (5)	C16—C17—H17C	109.5
C9—C6—C5	111.4 (5)	H17A—C17—H17C	109.5
C8—C6—C5	111.0 (5)	H17B—C17—H17C	109.5
C9—C6—C7	108.5 (5)		

Symmetry codes: (i) −*x*+2, −*y*+1, −*z*+1.
